# *Epigenetics & Chromatin *celebrates its first anniversary

**DOI:** 10.1186/1756-8935-2-13

**Published:** 2009-11-02

**Authors:** Steven Henikoff, Frank Grosveld

**Affiliations:** 1 Basic Sciences Division and Howard Hughes Medical Institute, Fred Hutchinson Cancer Research Center, Seattle Washington, USA; 2 Department of Cell Biology, and Department of Reproduction and Development, Erasmus MC - University Medical Center, Rotterdam, The Netherlands

## Editorial

*Epigenetics & Chromatin *published its first papers a year ago, together with an editorial in which we stated our aim to publish a high-quality journal with a broad scope. A year later we are happy to report that these aims are being achieved. Papers published so far represent a broad swath of research on chromatin-based processes and epigenetic mechanisms. We are also delighted with the quality and breadth of submissions from many of the leading laboratories in the field.

Among the papers in our inaugural issue was one from Elizabeth Blackburn and co-workers on the use of novel 4D imaging describing how human telomeres behave in vivo [1]. This landmark study demonstrating the extraordinary potential of modern imaging technology for following chromosome movements remains our most highly accessed paper. A year later, we are delighted to congratulate Dr. Blackburn for sharing the 2009 Nobel Prize in Physiology or Medicine, honoring her pioneering work on telomeres and telomerase. Telomeres have also been of great interest in the field of epigenetics and chromatin, with telomere position-effect and subtelomeric repeats providing important insights into the relationship between chromatin structure and gene silencing. Understanding the role of telomeres in disease continues to be an important area of research represented in *E&C *[2].

Here we review a collection of recent articles that we believe illustrate the scope and quality of articles published in *E&C*, which have also been collated to form a special anniversary print issue. The most popular model systems for epigenetic research are represented among them, including mice, yeast and flies. The articles range from studies of classical epigenetic phenomena, such as X-chromosome inactivation [3] and position-effect variegation (PEV) [4], to biochemical and biophysical approaches, such as chromatin complex purification [5] and atomic force microscopy [6]. Below, we discuss a few of these findings.

The classical phenomenon of PEV, the heritable "spreading" of the silent state into a gene by juxtaposition to heterochromatin, has intrigued geneticists for 80 years. But despite substantial progress in understanding the components of spreading, the mechanism has remained in doubt. In their *E&C *paper published in January, 2009 [4], Bas van Steensel and colleagues address the question of spreading by determining the precise pattern of binding by Heterochromatin-associated Protein 1 (HP1) in classical *Drosophila white *locus PEV mutants. Surprisingly, they find that the distribution of HP1 is highly inhomogeneous, and that the *white *gene is unusually sensitive to heterochromatic silencing. These findings led the authors to propose a model that reconciles some of the most puzzling features of PEV.

Pluripotency lies at the heart of reprogramming and stem cell biology, yet its molecular basis remains unknown. Mouse embryonic stem cells represent a favorite model for studying the relationship between epigenetics and chromatin, and this system was used by Azim Surani and colleagues to investigate the possible role of the ESET histone methyltransferase in regulating pluripotency. In their *E&C *paper published in October, 2009 [7], they describe a novel mechanism whereby regulation of pluripotency in the trophectoderm lineage is maintained, and perhaps established, via recruitment of sumoylated ESET by the Oct4 transcription factor. This connection between the transcription factor that regulates pluripotency, a chromatin regulator that methylates a key histone residue, and modification by SUMO ligation, provides an intriguing glimpse into the nuts and bolts of a key developmental process.

Ultimately, understanding the role of chromatin in mediating epigenetic inheritance requires a more complete description of the process that occurs behind the replication fork, where old nucleosomes are transmitted to daughter strands and new nucleosomes are assembled. However, little is known about this process in vivo, and as a result, the chromatin basis for epigenetic inheritance remains the subject of much speculation. In their *E&C *paper published in September, 2009 [8], Jessica Tyler and colleagues describe a novel strategy for investigating this process. Using an inducible yeast promoter that depends upon nucleosome depletion for initiation of transcription, they show that neither the presence of the activator nor ongoing transcription are required for inheritance of the nucleosome-depleted state. This elegant experiment argues against the simple notion based on phage genetics that rebinding by a transcription factor behind the replication fork is what maintains epigenetic inheritance in eukaryotes and reveals how little we truly understand about chromatin memory.

The tremendous progress that we have witnessed in the chromatin and epigenetics field exemplified by these studies has led to an increasing appreciation for the importance of chromatin-based processes in biological regulation. As a result, many researchers are finding that their work is becoming more competitive for space in high-impact journals. We recognize that in these cases a journal such as ours may not be the first choice for submission. Nevertheless, we think that by maintaining the high quality of published work, *E&C *will become increasingly attractive for publication of the most exciting research in the field. Among the advantages of publishing in *E&C *are that upon acceptance, a paper is immediately published online in preliminary form, highlighted on the *E&C *web site and indexed on PubMed. A particular advantage of online publication is that papers can be made more readable by including crucial data and procedures as part of the main body of the paper, rather than being relegated to supplements. As co-Editors-in-chief with long experience in this field, we exercise our judgment concerning the necessity for additional experiments proposed by referees that would otherwise delay publication of an exciting result and/or represent an unreasonable amount of work. Should the circumstances merit it, we are glad to consider accelerated publication of a paper submitted with decision letters and complete peer reviews from a high-impact journal. This policy was proposed and approved at a recent meeting of the *E&C *Editorial Board, and we hope that potential authors will consider this option as a way of achieving very rapid publication in a highly competitive arena.

As pleased as we are with the overall quality of manuscripts submitted to *E&C*, we still need to increase the number of submissions and subsequent publication in order for this venture to succeed. Therefore, when trying to decide where to send your work for publication, we ask that you take into account the high editorial standards of *E&C*, the breadth and excellence of our Editorial Board, the rapid publication upon acceptance, and the advantages of open access in reaching your intended audience. On behalf of the *E&C *team and the Editorial Board, we hope that you will consider our journal for submitting your work for publication.
